# Arsenic in Drinking Water and Lung Cancer Mortality in the United States: An Analysis Based on US Counties and 30 Years of Observation (1950–1979)

**DOI:** 10.1155/2016/1602929

**Published:** 2016-06-13

**Authors:** Hamid Ferdosi, Elisabeth K. Dissen, Nana Ama Afari-Dwamena, Ji Li, Rusan Chen, Manning Feinleib, Steven H. Lamm

**Affiliations:** ^1^Center for Epidemiology and Environmental Health, CEOH, LLC, 3401 38th Street NW, Suite 615, Washington, DC 20016, USA; ^2^Milken Institute School of Public Health, George Washington University, 950 New Hampshire Avenue NW, Washington, DC 20052, USA; ^3^Department of Pathology, Johns Hopkins University School of Medicine, 600 North Wolfe Street, Baltimore, MD 21287, USA; ^4^Center for New Designs in Learning and Scholarship, Georgetown University, 3520 Prospect Street NW, No. 314, Washington, DC 20057, USA; ^5^Johns Hopkins University Bloomberg School of Public Health, 615 N Wolfe Street, No. 5041, Baltimore, MD 21205, USA; ^6^Georgetown University School of Medicine, 3800 Reservoir Road NW, Washington, DC 20007, USA

## Abstract

*Background.* To examine whether the US EPA (2010) lung cancer risk estimate derived from the high arsenic exposures (10–934 *µ*g/L) in southwest Taiwan accurately predicts the US experience from low arsenic exposures (3–59 *µ*g/L).* Methods*. Analyses have been limited to US counties solely dependent on underground sources for their drinking water supply with median arsenic levels of ≥3 *µ*g/L.* Results*. Cancer risks (slopes) were found to be indistinguishable from zero for males and females. The addition of arsenic level did not significantly increase the explanatory power of the models. Stratified, or categorical, analysis yielded relative risks that hover about 1.00. The unit risk estimates were nonpositive and not significantly different from zero, and the maximum (95% UCL) unit risk estimates for lung cancer were lower than those in US EPA (2010).* Conclusions*. These data do not demonstrate an increased risk of lung cancer associated with median drinking water arsenic levels in the range of 3–59 *µ*g/L. The upper-bound estimates of the risks are lower than the risks predicted from the SW Taiwan data and do not support those predictions. These results are consistent with a recent metaregression that indicated no increased lung cancer risk for arsenic exposures below 100–150 *µ*g/L.

## 1. Introduction

Arsenic is a known human carcinogen, whether the exposure is by ingestion or inhalation. Arsenic ingestion by populations exposed to arsenic concentrations in drinking water of at least several hundred micrograms per liter (*μ*g/L) is associated with skin cancer, bladder cancer, and lung cancer [1]. A number of other sites have isolated positive findings, but skin, lung, and bladder cancer are consistently found to be increased with ingestion of high levels of arsenic [1]. The primary strong findings are in the Chilean studies where major parts of the population had average exposures of 600–860 *μ*g/L [2] and in the SW Taiwan study where major parts of the population had high exposures of 300–590 *μ*g/L and still higher exposures of ≥600 *μ*g/L [3].

Cancer risk estimates for lower levels have generally been derived by backward extrapolation of risk at high levels. Cantor has observed that “most estimates of risk at low and moderate levels of exposure (<200 *μ*g/L) have been based on extrapolation from ecological studies of populations exposed to higher levels” [4]. In February 2010, the US Environmental Protection Agency (US EPA) circulated a never-finalized draft toxicological review of inorganic arsenic which presented unit risks for US male and female lung cancer incidences [5]. These risks were derived from high median arsenic exposure levels (mainly 300–934 *μ*g/L) in SW Taiwan and were presented as being the risks expected to have been experienced in the US where the population generally has low arsenic exposure levels (3–59 *μ*g/L) [5].

While most epidemiological studies where drinking water arsenic levels are in the hundreds of micrograms arsenic per liter range do indicate a significant strong association for lung cancer [6], few studies report such risks in the lower range of <100 *μ*g/L. A number of lung cancer studies showed no significant increased risk below 100 *μ*g/L [7–11], while others did [2, 12, 13]. Other studies using either toenail or urine arsenic metrics found no increased risk in their low exposure groups [14, 15]. This study adds to the developing literature and provides more data points in the up to 60 *μ*g/L arsenic range than do any of the other studies.

The current study is analogous to the male bladder cancer ecological study of 133 US counties (1950–1979) with median groundwater arsenic levels in the 3–59 *μ*g/L range (measured in the period 1973–1998) [16]. The bladder cancer study found no arsenic-related increase in male bladder cancer mortality in that exposure range. The 95% upper-bound estimate of the risk was found to be lower than the risk predicted by the National Research Council (2001) but not that predicted by the US EPA (2001) [17, 18]. The NRC (2001) and US EPA (2001) risks had been based on Morales et al.'s (2000) dataset [19] for the 42-village study from SW Taiwan where median exposures ranged up to 934 *μ*g/L presented in the NRC (2001) Table  A10-1 [17–19].

This analysis of US counties lung cancer mortality expands upon that analysis of US counties bladder cancer mortality [16]. Specifically, it uses the same exposure and outcome data but now for lung cancer, for males and for females, and for a greater number of counties.

Further, we were able to obtain county-specific covariable information that had not been available ten years earlier. Finally, we were able to compare the 95% confidence limits based on the US observations and compared the upper-bound estimate with that predicted by the more recent US EPA [5, page 131].

## 2. Materials and Methods

This study is an ecological study with the goal of assessing the association between arsenic in drinking water and lung cancer. This study combined the county-specific median arsenic groundwater data from the US Geological Survey (USGS) [20] with the county-specific lung cancer mortality data for the period 1950 to 1979 from the National Cancer Institute (NCI) and US Environmental Protection Agency [21]. It was limited to those counties assessed by their state departments of health or of the environment to be solely dependent on groundwater for their drinking water supply, that is, having no surface water sources.

### 2.1. Exposure Measure

The exposure data come from the USGS National Water Information System [20] dataset that contained arsenic measurements from 67,440 wells with potable water. The data were collected in 1973–1998 and released in November 2001. The assessments of groundwater arsenic levels were made in the late 20th century and are assumed to reflect the groundwater arsenic levels back to the early 20th century. These are the earliest known national groundwater arsenic data.

USGS [20] developed a set of county-specific summary statistics of arsenic levels in potable groundwater well sources in *μ*g/L. The summary statistics included the median, mean, and maximum values, as well as 10th percentile, 75th percentile, and 90th percentile levels for each county with sufficient data—each determined by USGS. Counties with fewer than five wells within 50 km radius of the center of the county were considered to have insufficient data for the calculation of summary statistics. This analysis only includes counties which the USGS [20] considered to have sufficient number of well sources to develop county-specific summary statistics. For each well, the latest specimen was used. All of these measurements had been made using hydride generation.

USGS [20] assigned a representative median arsenic concentration to each county, and we have used those county-specific values. There is no breakdown of subpopulations within the counties, so the median arsenic concentration is assumed to represent the exposure of the entire population. Of the 2,277 counties, 1,594 had sufficient data for computing summary statistics with medians of 1 *μ*g/L to 59 *μ*g/L and 682 did not. In parallel to the 2004 report on US bladder cancer mortality and groundwater arsenic levels, selection was limited to the counties with a median of 3 *μ*g/L arsenic or higher (*N* = 268). State departments of health and of the environment were contacted to identify which of these counties were solely dependent on groundwater for their drinking water supply, that is, lacking surface water sources for drinking water. These data produced a set of counties (*N* = 186) that could be included in the analysis. The range for the median arsenic exposure of these 186 counties was 3–59 *μ*g/L arsenic.

### 2.2. Outcome Measure

The outcome data came from the NCI/EPA report on US County Cancer Mortality Rates and Trends, 1950–1979 [21]. The lung cancer data in the NCI/EPA 1950–1979 cancer mortality report was presented as counts and age-adjusted rates (adjusted to the 1970 US population) for each decade and separately for the four race- gender demographic groups—white males, white females, nonwhite males, and nonwhite females. Nonwhites included African-Americans, American Indians, and Asians. Such data existed for the US, each state, and each county in the 48 states that comprised the USA in 1950 (i.e., excluding Alaska and Hawaii which became states in 1959). Lung cancer mortality data that included at least one lung cancer death and an annual average age-adjusted death rate for each of the three decades—1950–1959, 1960–1969, and 1970–1979—was used as an inclusion criterion for the outcome of interest.

The Standard Mortality Ratio (SMR) for each study county served as the outcome metric. The SMR was calculated as the sum of the observed number of lung cancer deaths over the three decades divided by the sum of the expected number of lung cancer deaths by decade. The expected number was the number of lung cancer deaths that would have been expected if that county's population had the same mortality rate as did its state.

### 2.3. Covariables

Potential demographic county level covariables of education (percent of non-high school graduates), rural (percent of the rural), poverty (percent of those below 75% of the poverty level), and income (median household income) came from the 1970 US census data [22]. Education, rural, and poverty data were obtained through the National Historical Geographic Information System (NHGIS) [23]. Nativity (percent of cancer deaths to person born in state) came from US Vital Statistics 1979 [24]. County-average soil arsenic levels (including sediment) were available from the USGS National Geochemical Survey for all 173 counties [25] and were included as a proxy for dietary arsenic. County-specific smoking rates for males and females were available based on the 1996 Behavioral Risk Factor Surveillance System (BRFSS) data [26, 27]. The US Census Bureau provided the county population data [23].

### 2.4. Analytic Methods

A scatterplot of the county SMRs on the county median groundwater arsenic levels was visually examined to assess both linearity of relationships and the occurrence of disparate elements (outliers). Regression analyses were tested with both a linear and a quadratic term in order to assess for nonlinearity. The quadratic term to the model was not found to be significant, which excluded at least one form of a nonlinear relationship. Population-weighted least square regression analyses adjusted for the size of each population were conducted to reduce the effect of the wide range of population sizes across the counties. The 1960 white male and female populations were used to represent the 1950−1979 period as it was more representative of the prior years. Multiple linear regression analyses were conducted to determine which covariables were significantly related and to adjust for their effects. Statistical significance was based on a two-tailed *p* < 0.05. The multivariate analyses provide a sensitivity analysis with respect to the examined covariables.

Backward elimination strategy was used to select a final model based on the *p* = 0.05 significance level. The analysis began with all the variables included in the model and followed with sequential removal on the basis of statistical significance. The final model contained only those variables that each remained as statistically significant predictors.

Further, hierarchical linear regression analysis was conducted to assess the significance of the unique variance additionally contributed by the median groundwater arsenic level to the SMRs analyses. Regression analyses were conducted using Stata 11.0 [28], and scatter plots with trend lines were constructed using Excel [29]. As an alternative, a stratified linear regression relative risk analysis was conducted that provides a more granular look at the pattern of the SMR over the exposure range. The SMR for each stratum was the average of the SMRs for the counties in that stratum, and the exposure for each stratum was the population-weighted average of the median exposures for those same counties. The relative risks were calculated as the ratio of the SMR in the exposure strata divided by the SMR in the reference exposure level, a median exposure of 3.0 *μ*g/L.

Analysis was extended to determine the “unit risk,” that is, the increased lifetime risk of being diagnosed with lung cancer per lifetime average increased exposure to 1 *μ*g/L arsenic in drinking water. This unit risk for lung cancer and drinking water arsenic exposure was derived from the US data. As the “unit risk” is a linear function in concept and in use, the unit risk analyses have been conducted using arsenic level as a linear function. The maximum unit risk is the 95% upper confidence bound of the unit risk and is the maximum unit risk that is still compatible with the underlying data.

The coefficient (slope) for arsenic in the regression analyses was converted to a unit risk estimate in a series of steps. The national background 1950–1979 lung cancer mortality rates in the NCI/US EPA [21] report (46.8/100,000 PYs for US white males and 9.3/100,000 PYs for US white females) were converted to lifetime lung cancer mortality risks with the assumption of an average 70-year lifetime. This yielded lifetime lung cancer mortality risks of 0.0327 (46.8/100,000 *∗* 70 = 0.0327) for males and of 0.0065 (9.3/100,000 *∗* 70 = 0.0065) for females.

The baseline lifetime mortality risk at zero arsenic level in drinking water for the study population was the product of the lifetime mortality risk and the intercept from the regression model. The lifetime mortality unit risk for arsenic exposure was the product of the baseline lifetime mortality risk at zero arsenic level and the coefficient (slope) for the median arsenic exposures (*μ*g/L).

The lifetime mortality unit risk for arsenic exposure was then converted to a lifetime incidence unit risk for arsenic exposure using the SEER data (1975–2010) [30] lung cancer incidence to mortality ratio of 1.12 for males and 1.34 for females. The total factor for converting the coefficient (slope) for lung cancer mortality to a lifetime incidence unit risk was 0.0367 (0.0327 *∗* 1.12 = 0.0367) for males and 0.0087 (0.0065 *∗* 1.34 = 0.0087) for females. The maximum lifetime incidence unit risk for arsenic ingestion (per *μ*g/L) was the largest unit risk that would still be consistent with the underlying data (95% upper confidence bound (95% UCB)). The maximum unit risk (Max UR) for lifetime lung cancer incidence per *μ*g/L arsenic ingested was the product of the intercept, the 95% UCB, and the conversion factor.

The maximum URs derived here from the US data for US males and females were compared with the lung cancer unit risks predicted in US EPA [5] for the US male (1.9*E* − 4) and for the US female (4.8*E* − 4) populations which had been derived from the southwest Taiwan data [19]. The comparison assesses whether the unit risks predicted from the SW Taiwan data are compatible with the observed US data. Sensitivity analyses were also performed to determine whether the wide range of county characteristics (numbers of wells, population, and area) had a significant effect on the outcome.

Data on nonwhites had not been included in the main analysis both because the small number of eligible counties (28 counties for males and 13 counties for females) made them unlikely to be representative of the nonwhite US population and because the county covariables (education, poverty, median household income, and rural) were unlikely to have been applicable to the nonwhite population. Because of the small number of counties meeting the outcome criterion for nonwhite males or females, the analysis in this report was limited to the data on white males and females.

## 3. Results

Of the 186 counties that met the exposure criteria, 173 counties (173/186 = 93%) met the outcome criteria for white males, 133 for white females (133/186 = 71%), 27 for nonwhite males (27/186 = 15%), and 13 for nonwhite females (13/186 = 7%). The 173 counties with white male data were located in 26 states and included the 133 counties with the white female data. The analyses presented here have been restricted to the white population. The median arsenic levels for the counties with sufficient white lung cancer data ranged from 3 *μ*g/L to 59 *μ*g/L. The slopes were nonsignificant and negative.

Similar analyses were performed for the counties with sufficient nonwhite data. The median arsenic levels for the counties with sufficient nonwhite lung cancer data ranged from 3 *μ*g/L to 17 *μ*g/L. The slopes, also, were nonsignificant and negative. For those counties with sufficient white and nonwhite data for males and females, the slopes for white and nonwhite male lung cancer mortality were similar to each other, as were the slopes for white and nonwhite female lung cancer mortality (not shown).

Table 1 shows the descriptive statistics for covariables as well as those for the dependent variables for lung cancer (white male SMR and white female SMR) and for the independent variable (median arsenic exposure). The median arsenic levels ranged from 3 *μ*g/L to 59 *μ*g/L based on measurements from 7,669 wells in 173 counties with an average of 44 wells per county and a range of 5 to 510 wells per county.

**Figure 1:**
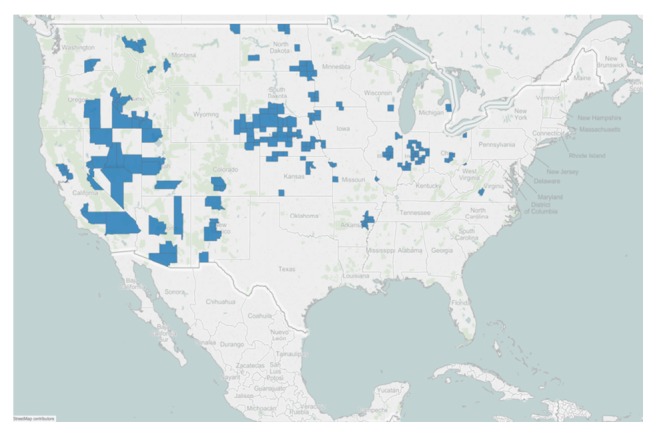
The distribution of study counties across the contiguous 48 states.

The study counties are distributed generally across the contiguous 48 states except for the south and northeast sections (Figure 1). Nevada and Idaho in the west and South Dakota and Indiana in the north central regions had the most counties.

### 3.1. Simple Regression Analysis

The male SMRs ranged from 0.42 to 1.37 with extreme outliers of 1.79 and 2.10. The two counties (Deer Lodge County, Montana; Storey County, Nevada) with extreme outliers (*Z* ≥ 4.0) were excluded from the analysis. Both Deer Lodge County (arsenic = 59 *μ*g/L) and Storey County (arsenic = 6 *μ*g/L) had markedly excess lung cancer mortality for white males, but not for white females, suggesting an occupational etiology. Deer Lodge County, Montana, is the site of the Anaconda copper smelter, where studies have shown that respiratory cancer excess in the male population was due to high levels of arsenic inhalation [31, 32]. Storey County, Nevada, was a retirement area for underground gold miners, a group with known increased risk for respiratory cancer [33]. This background provides an explanation for the extreme rates which were observed only for the males.

**Figure 2:**
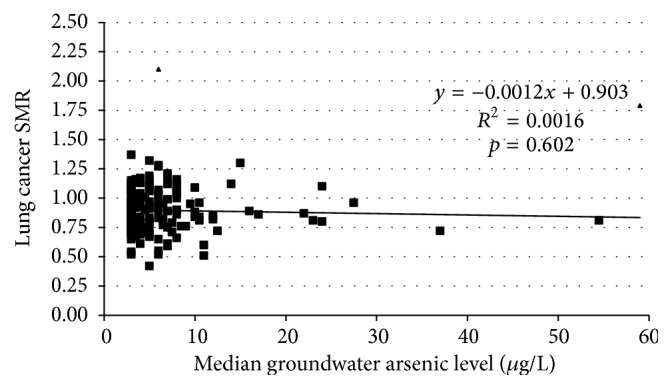
White male lung cancer SMRs (1950–1979) by median groundwater arsenic level (*μ*g/L). Triangle—outlier male SMR (Deer Lodge County, Montana, and Storey County, Nevada).

The final 171 county analytic dataset for the males contained 33,304 lung cancer deaths, 78 million (78,172,710) person-years of observation, and a 1960 white male population of 2.6 million (2,605,757).  Figure 2 shows the scatter plot of the white male lung cancer SMRs for the 171 counties analyzed with respect to their median groundwater arsenic levels. The SMR data for males regressed on the median groundwater arsenic levels showed a *y*-intercept at zero arsenic of 0.903 (95% CI, 0.864 to 0.942) and a negative but nonsignificant (*p* = 0.602) slope of −0.0012 (95% CI, −0.006 to +0.003). Simple linear regression on the arsenic exposure data explained less than 0.2% of the variance of the SMR data for males (*R* ^2^ = 0.0016).

**Figure 3:**
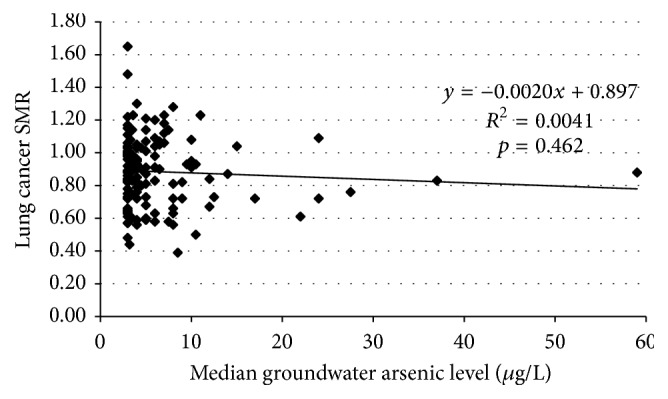
White female lung cancer SMRs (1950–1979) by median groundwater arsenic level (*μ*g/L).

The female lung cancer SMRs were found to have a range of 0.36 to 1.65, with no extreme outliers. The final 133 county analytic dataset for the females contained 7,778 lung cancer deaths, 76 million (75,696,150) person-years of observation, and a 1960 white female population of 2.5 million (2,523,205).  Figure 3 shows the scatter plot of the white female lung cancer SMRs for the 133 counties analyzed with respect to their median groundwater arsenic level. The SMR data for females regressed on the median groundwater arsenic levels showed a *y*-intercept at zero arsenic of 0.897 (95% CI, 0.847 to 0.947) and a nonsignificant (*p* = 0.462) negative slope of −0.0020 (95% CI, −0.007 to +0.003). Simple linear regression of the arsenic exposure data explained about 0.4% of the variance of the SMR data for females (*R* ^2^ = 0.0041).

Analyses that excluded the two highest arsenic levels, that is, of data for arsenic level < 30 *μ*g/L, yielded slopes of 0.000 for males (*p* = 0.91) and for females (*p* = 0.36) and with little explanatory power (*R* ^2^ < 0.004). The maximum unit risks are calculated to be 2.27*E* − 04 and 3.64*E* − 05 for males and females, which reject the EPA predicted unit risk at the 90th and 95th percentiles, respectively.

Sensitivity analyses were carried out with respect to county characteristics of number of wells, population, and area. Analyses were restricted to eliminate the more extreme values of few wells, low population, and large area, each by one to two orders of magnitude. Sensitivity analyses were conducted on number of wells per county (≥5 to >50), population per county (>0.4 K to >5 K), and square miles per county (<25,000 to <1,500). In all cases, the slopes were similarly negative, and the intercepts were less than 1.00. Removal of the more extreme values had no effect on the analytic results. The slope was independent of data density (i.e., wells per square mile) with the slope and intercept for counties above the median (0.0011) and below the median (0.0013) being essentially equivalent, an issue raised by Ryker [32].

### 3.2. Multiple-Regression Analyses

Each white male lung cancer model contained data from 171 counties in 26 states. The arsenic coefficient (slope) was negative in the unadjusted model and positive in the adjusted models. Rural was the only significant variable in the arsenic model with all seven covariables. The unit risk was calculated to be −4.44*E* − 5 in the unadjusted model, 1.62*E* − 5 in the model with all covariables, and 1.05*E* − 5 in the model with the significant covariable (rural). A negative unit risk is interpreted as equal to zero. The maximum unit risk was 1.05*E* − 4 in the unadjusted model, 1.47*E* − 4 in the model with all covariables, and 1.72*E* − 4 in the model with the significant covariable. Expansion of the adjusted model to include all seven covariables nearly doubled the explanatory power from 0.072 to 0.133. The maximum unit risk in each of the three models was less than the US EPA [5] predicted unit risk of 1.9*E* − 4, rejecting it by a factor of 10–80% (or an average of 40%).

A model for male lung cancers with all seven covariables but without arsenic (not shown) as an explanatory variable had an *R* ^2^ of 0.1329, only slightly less than the *R* ^2^ = 0.1332 of the model with all seven covariables and arsenic. This indicated that arsenic increased the explanatory power by less than 0.05%. Hierarchical analysis showed a *p* value of 0.808 for the arsenic variable.

Each female lung cancer model contained data from 133 counties in 25 states. The arsenic coefficient (slope) was negative in all three models. Poverty was the only significant variable in the arsenic model with all seven covariables. The unit risk was also negative in all three models and statistically indistinguishable from zero. The maximum unit risk was 2.62*E* − 5 in the unadjusted model, 3.32*E* − 5 in the model with all seven covariables, and 2.56*E* − 5 in the model with the significant covariable (poverty). Expansion of the adjusted model to include all seven covariables nearly quadrupled the explanatory power from 0.0246 to 0.0888. The maximum unit risk in each of the three models was less than the US EPA [5] predicted unit risk of 4.8*E* − 4, rejecting it by a factor of 14–19-fold.

A model for female lung cancers with all seven covariables but without arsenic (not shown) as an explanatory variable had an *R* ^2^ of 0.0876, only slightly less than the *R* ^2^ = 0.0888 of the model with all seven covariables and arsenic. This indicted that arsenic increased the explanatory power by only 0.0012 (i.e., 0.12%). Hierarchical analysis showed a *p* value of 0.682 for the arsenic variable.

### 3.3. Stratified Analysis

The lung cancer mortality data can also be aggregated into a stratified analysis (Table 2). This is analogous to the previously published male bladder cancer mortality data [16].

With a single exception, each of the SMRs is less than 1.00, and many of them are statistically significant (i.e., 95% UCL < 1.00). The SMRs are fairly consistent at about 0.90, probably reflecting that most of the counties had the lung cancer risk pattern of rural counties rather than those of the areas that were urbanized with industrial and environmental exposures and higher smoking rates.

**Figure 4:**
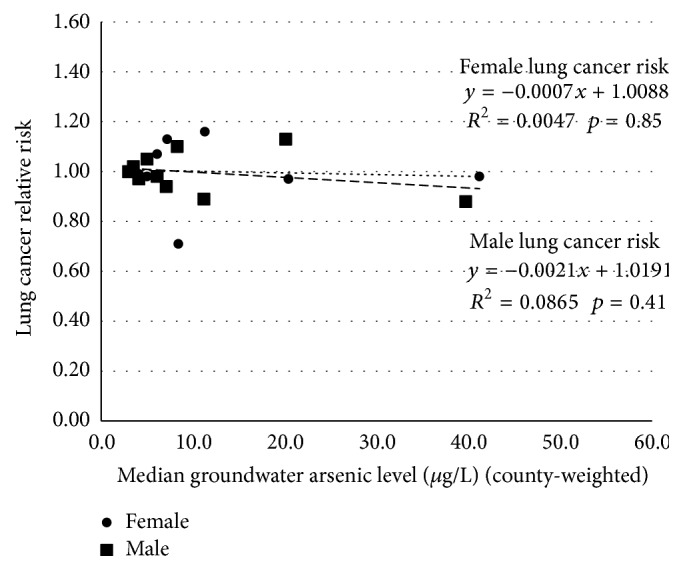
Lung cancer relative risks regressed on county-weighted median groundwater arsenic strata (*μ*g/L).

Regression of the relative risks on the average of the median groundwater arsenic levels in each stratum indicated a consistent pattern (Figure 4). The relative risks hover around 1.00. Further aggregation of the data into strata of 3.1–9.9 *μ*g/L and 10.0–59.9 *μ*g/L with 3.0 *μ*g/L as the reference populations yielded relative risks of 1.01 and 0.98 for males and 1.01 and 0.97 for females, showing no increase in risk across the observed exposure range.

For both females and males, the slopes were slightly negative (−0.001 and −0.002, resp.), and their *p* values (0.846 and 0.345, resp.) were not statistically significant. Visually, the slope for the female relative risks looks zero and that for the male relative risks nearly so. The maximum unit risks, 1.08*E* − 04 for males and 2.62*E* − 05 for females, are calculated to be less than the US EPA [5] predicted unit risks of 1.9*E* − 04 and 4.8*E* − 04, respectively. It is noteworthy that in spite of having consistently low SMRs the relative risk analysis demonstrates that within this exposure range the lung cancer risk is not dependent upon the drinking water arsenic level. The stratified data yield maximum unit risks that reject the EPA predicted risk by factors of 40% and 7-fold, respectively.

The results from the regression of the stratified data (Figure 4) are similar to those from the regressions of the disaggregated data (Figures 2 and 3). The stratified analysis, however, shows that the pattern is consistent across the exposure range and not the consequence of a significantly low finding at the high end of the exposure range.

## 4. Discussion

This is the first nationwide US study of the relationship between lung cancer mortality and the level of arsenic in the local drinking water supply. It shows no arsenic-related increase in lung cancer mortality (1950–1979) for US counties exclusively dependent on groundwater with median arsenic concentrations in the range of 3–59 *μ*g/L for their drinking water supply.

This study is of special importance because it provides an opportunity to evaluate the US EPA [5] male and female lung cancer risk estimates for arsenic ingestion. US EPA [5, page 131] had developed unit risks for arsenic ingestion (lifetime lung cancer incidence risk per *μ*g/L arsenic ingested) of 1.9*E* − 4 for US males and 4.8*E* − 4 for US females, which were based on the southwest Taiwan data. In contrast, our analyses which are based on US data found the unit risk to be 1.62*E* − 5 for US males and a negative value (−8.69*E* − 6) for US females in the fully adjusted model. Further, the maximum unit risks for arsenic ingestion (lifetime lung cancer incidence risks per *μ*g/L arsenic) were found to be 1.47*E* − 4 for US males and 3.32*E* − 5 for US females in the fully adjusted models. Thus, the analysis of the southwest Taiwan data developed in US EPA [5] significantly overpredicted the lung cancer unit risks as experienced in the US.

*Comparison with Analysis of SW Taiwan Data*. The US EPA [5] lung cancer risk estimates were derived using lung cancer mortality data from the Morales et al.'s dataset [19] for the Wu et al.'s [3] study of the 42 villages in the Blackfoot-endemic area of southwest Taiwan. These villages had median arsenic exposures that ranged from 10 *μ*g/L to 934 *μ*g/L and a population-weighted average exposure of 305 *μ*g/L. In contrast, the US study of 171 and of 133 counties had a median arsenic exposure range of 3 *μ*g/L to 59 *μ*g/L and a population-weighted average exposure of 5 *μ*g/L. The analysis in this study demonstrates that the risk estimated from a population averaging 305 *μ*g/L does not predict the risk experienced by a population averaging 5 *μ*g/L. Similarly, an analysis of the southwest Taiwan data excluding the reference population demonstrated that the risk estimated for a population that included those with median, mean, or maximum exposures above 200 *μ*g/L did not predict the risk among those with exposures below 100 *μ*g/L [34].

The US study and the SW Taiwan study are both ecological studies that use local well water arsenic data and that lack individual exposure data; thus, results in each should be interpreted cautiously. In both cases, the analyses have been based on the median values. The median values do not incorporate the range of measurements but do represent a measure of their central tendency. In neither case is there information on what proportions of the populations used waters of different arsenic levels, so it is not possible to calculate population-weighted means, and medians have had to suffice as the representative exposure measure.

Both our analyses and those of the EPA were conducted in the 21st century and are based on data from the late 20th century. Our period of mortality is 1950–1979 and that of the EPA is 1973–1986, so they are temporally similar. While the most relevant exposure data for both studies would have been of drinking water arsenic levels from the late 19th century to the late 20th century, the earliest extant data are from 1973 to 1998 for the US data and from 1962 to 1964 for the SW Taiwan data. The USA counties were ascertained in 2000-2001 to be free of surface waters as drinking water sources, thus the assumption that the groundwater sources represented the actual exposure in not confounded with unascertained surface or piped water sources. In contrast, the well sampling for the SW Taiwan villages was in 1962–1964, some years after they had begun to receive surface or piped water in the 1950s. Thus wells that had been turned off or were not extant during 1962–1964 would not have been sampled, casting some doubt as to whether the sampled wells indeed represented the historic well water sources for the individual villages.

The US counties had a minimum of five sources and a median of 17 sources. Most of the villages in the SW Taiwan villages had measurement from a single source (22 of 42 villages) and the median was 2 sources. The US study was restricted to those counties that only used groundwater as their drinking water sources. Bottled water consumption at that time would not have been a confounding exposure as consumption in the 70s was only 5% of the consumption rate in the 2000s [35]. While clean water was brought to SW Taiwan villages starting in about 1956–1959, the SW Taiwan study had assumed that the wells sampled in 1964–1966 comprised all the wells that the villages had ever used and that no bottled water was used.

While the US study includes county-specific covariables, the SW Taiwan study had no village-specific covariables but instead assumed uniformity across all villages for all variables other than well water arsenic levels. The US study includes county data on standard demographic covariables—education, rural, poverty, and income—and the best available data on specifically relevant covariables—soil arsenic, nativity, and smoking. This would have brought in some consideration of arsenic exposures from smoking and from the consumption of locally grown foods but not of other dietary sources, such as rice. The US analysis here is limited to the white population, while the SW Taiwan population is Taiwanese. The SW Taiwan results were then transposed to a mainly white population. 

*Arsenic Exposure Data*. National US data on groundwater arsenic levels did not exist until 1973–1998. Their use here assumes that there was little change in the levels over the earlier decades. That assumption is not unreasonable. The USGS [20] did assess whether groundwater arsenic levels varied with time and concluded that “there probably is no relation between arsenic concentration and time for most wells.” Their time interval may have extended up to two decades. Similarly, a study in Argentina reported that groundwater arsenic levels showed a high degree of consistency over a 50-year period [36].

While we have no US data on the steadfastness of groundwater arsenic levels over a half-century or more, we are not aware of any additional data from elsewhere that might examine that. D'Ippoliti et al. from Italy have argued that arsenic concentrations can be assumed to be stable where “arsenic contamination in groundwater (is) due to natural underlying geological processes and (in) the absence of any arsenic mitigation intervention” [13]. That may be applicable also for the US. The counties in this study were selected because they had not developed any surface water drinking water sources through the year 2000.

The USGS [20] dataset on groundwater arsenic levels in US counties included measures for the median, the mean, and the maximum levels, in addition to the 10th, 75th, and 90th percentiles. We used the median level as the measure of central tendency as the USGS had concluded that the median level represented “the true center of the data” and avoided the biased effect that the skewed distribution had on the mean level [37]. We also conducted analyses using the 10th, 75th, and 90th percentiles as well as the mean and the maximum. In those models, all coefficients were nonsignificant and negative for both male and female lung cancers with the exception of maximum arsenic which was nonsignificant and positive (not shown). 

*Nonwater Sources of Arsenic*. Food and soil provide additional alternative media for arsenic exposure. Again, data were not available prior to recent years. Xue et al. [38] showed for the USA an average dietary inorganic arsenic intake of 2 *μ*g/day using 2003-2004 NHANES data. Similarly, Kurzius-Spencer et al., using the 2003-2004 NHANES data as well as two datasets from Arizona (1995–1997 NHEXAS and 2006-2007 BAsES), reported geometric mean dietary inorganic arsenic intakes of 6–8 *μ*g/day [39, Table 3]. Further, Tao and Bolger [40, 41], using the 1991–1997 FDA Total Diet Study for Market Basket data, reported an average total inorganic arsenic intake of less than 10 *μ*g/day for most people in the US. We have run the models with assumptions of either 2 or 10 *μ*g/day arsenic from diet together with an ingestion of 1 or 2 liters of water per day and found no effect from the assumptions for dietary contribution.

Xue et al. [38] had found that vegetables were the primary contributor (24%) to dietary inorganic arsenic. We presumed that locally grown vegetables were a greater part of the diet in the earlier years and may have reflected local soil arsenic levels. Therefore, we used soil arsenic levels as a proxy for dietary inorganic arsenic. Two sources for county-averaged levels of arsenic in the soil—the USGS National Geochemical Survey [25] and USGS Geochemical and Mineralogical survey [42]—were used as covariables. No effect on the coefficient (slope), *p* value, or 95% upper confidence limit of the median groundwater arsenic variable in the model was found. We found no correlation with soil/sediment values and groundwater arsenic levels. While a recent US study had reported an association between soil/sediment arsenic values and lung cancer [43], we were unable to confirm it using either their data or ours.

Cigarettes are a known source of arsenic exposure. Agency for Toxic Substance and Disease Registry [44] reports that tobacco contains an average arsenic concentration of 1.5 *μ*g/cigarette and yields a mainstream dosage of 0–1.4 *μ*g/cigarette (midrange 0.7 *μ*g/cigarette). The American Lung Association [45] reports that the average daily consumption of cigarettes per adult in 1996 was about 7 cigarettes per day. This would indicate a daily average consumption of about 5 *μ*g arsenic per day from cigarettes in the year for which we give smoking prevalence. This 5 *μ*g/day dosage would fit within the 2–10 *μ*g/day nondrinking water sources of arsenic in the calculations above. 

*Cigarette Smoking*. Cigarette smoking may contribute to the risk both as an additional source of arsenic exposure and for its effect on lung cancer risk. Smoking data, and in particular county-specific smoking data, were not available until the late 1990s. County-specific, gender-specific smoking rates are now available for the years 1996–2012 from the Behavioral Risk Factor Surveillance System (BRFSS) conducted by the Centers for Disease Control and Prevention [26, 27]. We have used the 1996 data, as they are the earliest available and closest to the time period of the observed mortality, and have used the assumption that the distribution of the 1996 county smoking rates reasonably reflects the relative distribution of the county-specific rates some decades earlier. We have used the above data to test this assumption by asking how well the smoking rates for 1996 and 2012 (an interval of 17 years) correlate. We found the correlation coefficient for county smoking rates over that 17-year interval to be 0.85 for males and 0.81 for females. Thus, we hold that it is not unreasonable to assume that the 1996 smoking rates distribution also reflects the county-specific smoking rates of previous decades. The US smoking rates were generally about 25% during the years of the BRFSS and about 40% during the years of the mortality observation [46]. We have assumed that, in spite of the general drop in the absolute smoking prevalence, the relative county-specific smoking prevalence has remained the same or similar.

ATSDR [44] noted that only with high levels of arsenic in drinking water did cigarette smoking increase the occurrence of lung cancer, citing cases of BFD disease in SW Taiwan [47] and exposures with very high arsenic levels [48]. Tsuda et al. [48] found an increased risk among smokers with exposure of ≥1,000 *μ*g/L but not at the lower exposure of 50–990 *μ*g/L. The Putila [43] analysis of soil/sediment arsenic as a risk factor for lung cancer showed that the odds ratio for arsenic (1.004) did not differ whether smoking was or was not included in the analysis. Further, no significant interaction between smoking and arsenic exposure has been found at exposure levels below 90–100 *μ*g/L [8, 10]. Additionally, Gebel et al. [49] reported that tobacco smoking did not have any influence on the contents of arsenic in urine or hair, and Demir et al. [50] reported no association between arsenic levels in lung cancer tissues and smoking status. Our observation of no association between lung cancer and smoking prevalence is consistent with the rest of the literature at exposures of less than 60 *μ*g/L arsenic. 

*Natality*. Both the previous bladder cancer study [16] and the SW Taiwan study [3] implicitly assumed that individuals were locally resident throughout most of their lives and at the time of their death. We have sought information on geographic mobility for the US counties. One metric that might have been used was the census data on the proportion of current residents that had been born in that place. However, those data are overweighted for the very young. As an alternative, we developed a metric using place of birth information on the 1979 US Vital Statistics public access data file for those who died from cancer (median age 62). We used as our metric the proportion of county cancer deaths that occurred to residents who were born in that same state. This metric was thus weighted to the age-group of interest. We observed that on average about 65–68% of the county residents dying from cancer had been born in the same state, suggesting that migration into the state may not have been such a major influence on the county cancer mortality rates. 

*Demographic Measures*. County-specific demographic variables on education, rural, poverty, and income were obtained from the 1970 US census data. Thus, these data were contemporary with the period of mortality observation. The only variables that were significant in the multivariable analyses were rural in the analyses of male lung cancer and poverty in the analyses of the female lung cancers. 

*Limitations*. Despite our finding that no arsenic-related increase in lung cancer mortality for US counties is exclusively dependent for their drinking water supply on groundwater with median arsenic concentrations in the range of 3–59 *μ*g/L, there are potential limitations to our study. The major limitations are that this is an ecological study and that the county-specific exposure variable and covariable information are generally for time periods after the period of mortality (1950–1979). Such data for earlier time periods do not exist.

The first major limitation of this study is that, a characteristic of ecological studies, per se, all measures are aggregate data rather than individual data and thus susceptible to the ecological fallacy. Further, the exposure metric (median groundwater arsenic concentration for the county) is a composite measure that does not express the variability in within-county exposure levels. The working assumption is then that all residents of the county were exposed to the median arsenic level for that county. The outcome measure (relative lung cancer mortality risk) has considered age, sex, race, state, and decade of death.

These analyses are based on 1950–1979 mortality data with exposure and covariable data from the mid-1970s to the mid-2010s. In order to deal with regional variations in diagnostic patterns, the county-specific risks were calculated relative to the risks for their individual states. An alternative analysis might have been to match counties with other counties of similar urban/rural status. That, however, would have required a number of selection assumptions that would raise their own issues. A second alternative might have been to use a more recent lung cancer data base. That alternative is being pursued so that its findings can be compared with these along with the then-current regulatory analyses.

The second major limitation of this study relates to the absence of temporal concurrence of the exposure information and the covariables. The groundwater arsenic data for US counties were only measured at the end of the 1900s and have to be presumed to have not changed significantly or relatively since the beginning of the 1900s. There are no data on which to assess that presumption and analytic methods at that time would have been insensitive to the exposure range here.

Similarly, regarding the cigarette smoking prevalence, we are well familiar with the marked changes nationally over the decades of the 20th century but we can only assume that the relative prevalence has not grossly changed in the second half of the 20th century, which we recognize as a weak but necessary assumption. For the other covariables, the earliest available data have been used.

The limitations of the SW Taiwan study that underlies the US EPA Toxicological Report [19] are generally similar to those of the US counties study. However, covariables (other than age and gender) were not obtained but were assumed to not have varied across the villages or to have been different for the regional population. No smoking information was obtained. 

### 4.1. Toxicological Model

Cohen et al. reviewed the toxicological literature evaluating the carcinogenicity of inorganic arsenic. They provided evidence for a mode-of-action (MOA) that involved the formation of reactive trivalent metabolites that interact with critical sulfhydryl groups, thus leading to cytotoxicity and regenerative cell proliferation [51]. They state that this MOA implies a nonlinear, threshold dose-response for both noncancer and cancer endpoints. Other MOAs might include genotoxicity and/or oxidative stress. US EPA notes that arsenic and its metabolites have not been found to induce gene (point) mutations and that a number of steps in the cytotoxicity-cell regeneration model may be stimulated by oxidative damage [5, page 99]. Snow et al. [52] had suggested a hermetic (biphasic) response at low concentrations, to which US EPA observed that “some low-dose effects (e.g., increased DNA repair) may be protective of carcinogenesis (and that) other effects (e.g., cell proliferation or telomerase activation) may … (at higher doses) permit mutant cells to survive by preventing cellular senescence and death and … (thus enhancing) arsenic's cancer-promoting capacity” [19, page 100].

In analyses on the SW Taiwan data that we have published elsewhere [53, 54], we have demonstrated high risks of both bladder and lung cancers with high levels (200–1,000 *μ*g/L) of arsenic in the drinking water. In contrast, our analyses of the bladder and lung cancer data for the villages in SW Taiwan with low levels (<100 *μ*g/L) demonstrated no exposure-related increased risk of either bladder or lung cancer in the 10–100 *μ*g/L exposure range. Similar to our findings for the SW Taiwan villages with low levels of arsenic in the drinking water, we found no exposure-related increased risk of bladder cancer mortality in the US county data [16] and now we demonstrate the same for lung cancers in the US mortality data.

Other recent epidemiological studies show similar results at exposures of <100 *μ*g/L as does this ecological study. A recent case/control study from western USA (California and Nevada) reported lung cancer odds ratios of 0.75 and 0.84 for exposures of 11–84 *μ*g/L (5-year average, 10-year and 40-year lag) compared to ≤10 *μ*g/L [9]. Additionally, a recent case/control study from Chile with individual exposure levels all at <100 *μ*g/L and determined over a 65-year period similarly found no significant increased risk for lung cancer across exposure tertiles (<10.1 *μ*g/L, 10.1–59.9 *μ*g/L, and >59.9 *μ*g/L), whether lagged 5 years or 40 years (Web Table 4) [11].

The fact that the data from all these studies are consistent with the threshold model is intriguing, since recent toxicological findings indicate that arsenic carcinogenicity may follow a two-step process with cytotoxicity being followed by cellular proliferation and a no-effect level being observed [51].

A variety of studies are beginning to show other toxicological effects related to mechanisms occurring with arsenic exposures in the hundreds of micrograms per liter but not below. Niedzwiecki et al. [55] found significant decreases in global methylation of DNA in peripheral blood monocytes only at exposure of greater than 300 *μ*g/L in the drinking water in Bangladesh. Similarly, Karakulak et al. [56] found that aortic elasticity parameters in arsenic-exposed workers diminished only when urinary arsenic levels exceeded about 150 *μ*g/L.

### 4.2. Risk Modeling

This risk modeling has used a linear model both because that is the underlying assumption of a unit risk and because an added quadratic term was not statistically significant (*p* value of 0.636 for males and 0.555 for females). The unit risk is, by convention, consensus, and usage, restricted to be nonnegative, that is, either zero or positive. The regressions above yielded negative coefficients and negative best estimates of the unit risk. In no case was the finding statistically significant. Thus, it is reasonable to conclude from this modeling that the lung cancer unit risks in this range of exposure (3–59 *μ*g/L) are indistinguishable from zero, an observation consistent with the threshold model. The 95% upper confidence limits of these unit risk estimates based on the US data were positive and were less than the lung cancer unit risks predicted in the US EPA [5] model based on the southwest Taiwan data. Thus, these data are inconsistent with the US EPA [5] linear no-threshold model that significantly overpredicted the lung cancer risks for the US population from the ingestion of drinking water containing arsenic in the 3–59 *μ*g/L range.

In contrast, these data are consistent with the results of a more recent systematic review and metaregression analysis of lung cancer risk and inorganic arsenic in drinking water which found no increased lung cancer risk at arsenic exposures below 100–150 *μ*g/L [6]. The metaregression analysis had found similar results for ecological studies (such as this one) and for nonecological (case-control and cohort) studies. Although ecological studies can be susceptible to the ecological fallacy, it is interesting to note that the analytic results and model fit from the ecological studies were equivalent to those from the nonecological (i.e., epidemiological) studies.

The US EPA [5] model considered a number of covariables or additional risk factors and then assumed that the exposures were similar across all the villages and thus differentially nondistinguishable. We have considered seven relevant covariables for which county-specific values were available. We have included them in our analyses. The use of the standard model that included the seven covariables for male lung cancers yielded a fit with *p* = 0.003, a positive unit risk, and a positive maximum unit risk which was less than the US EPA [5, page 131] predicted risk. The model explained ~13% (*R* ^2^ = 0.1332) of the variability in the SMR for males. The use of the standard model that included the seven covariables for female lung cancers yielded a fit with *p* = 0.160, a negative unit risk, and also a positive maximum unit risk which was less than the US EPA [5] predicted risk. The model explained ~10% (*R* ^2^ = 0.0888) of the variability in the SMR for females. When the standard model was restricted to inclusion of the significant covariables (rural in the male model and poverty in the female model), the explanatory powers were lower by a two- to fourfold factor but the maximum unit risks still were lower than those predicted by the US EPA model.

The observation is that for each linear model the best estimate of the slope over this exposure range of 3–59 *μ*g/L arsenic is indistinguishable from zero and a relative risk that is not greater than 1.0. The same was true when the exposure range was limited to 3 *μ*g/L to <30 *μ*g/L. This is in contrast to the significantly positive slope and relative risks for lung cancer observed in other studies where the exposures are in the hundreds of *μ*g/L arsenic. Taken together, these analyses suggest either a j-shaped, sublinear, or hormetic curve or alternatively a hockey-stick, or threshold, model. These data are inadequate to distinguish between these two models. For the most part, those analyses which in a univariate analysis show a statistically significant negative slope or reduced relative risk show in a multiple variable regression slopes that are indistinguishable from zero or relative risks indistinguishable from 1.0. Nonetheless, these data, showing no increased lung cancer risks with exposures below 60 *μ*g/L, are sufficient to exclude the lung cancer unit risks proposed by the linear nonthreshold model presented in US EPA [5, page 131]. Further, the WHO (2001) JECFA1 report [57], using the 5-stratum NE Taiwan lung cancer data of Chen et al. [8], found for a variety of models a* de minimis* risk range for the BMD_0.5_ equivalent to 59–102 *μ*g/L. Thus, our findings are quite consistent with those of other studies and analyses that have examined this* exposure* range.

### 4.3. Summary

In summary, analysis of the 1950–1979 US lung cancer mortality data for US counties that were solely dependent on groundwater for their drinking water sources found no association between lung cancer mortality and median groundwater arsenic level over the exposure range of 3 to 59 *μ*g/L arsenic. Almost all of the dose-response slopes in both the simple and multiple linear regression models were negative, and all were statistically indistinguishable from zero. These US county lung cancer mortality data do not demonstrate an increase in lung cancer risk with respect to median groundwater arsenic levels over the exposure range of 3–59 *μ*g/L arsenic. The upper-bound estimates of the risks, examined in a variety of models, were lower than the risks that had been predicted from the SW Taiwan data and do not support those predictions.

## Figures and Tables

**Table 1:** Descriptive table for variables in analysis of lung cancer mortality risk for white males and white females in study counties with median groundwater arsenic level.

	Units	Mean	Median	Range
Dependent variable				
SMR (WM)	—	0.9	0.89	(0.42–1.37)
SMR (WF)	—	0.88	0.89	(0.39–1.65)

Independent variable				
Median arsenic	*µ*g/L (ppb)	6.6	4.5	(3.0–59.0)
exposure				

Covariables				
Education	Percent < HS	47.3	46.8	(26.0–74.0)
Rural	Percent of adults	68	67	(5.0–100.0)
Poverty	Percent < 75th percentile	11	9	(3.0–36.0)
Income	Median income ($)	21,789	21,088	(11,151–37,669)
Soil/sediment	mg/Kg (ppm)	7.72	6.95	(1.23–30.95)
State born (M)	Percent of Ca deaths	57	65	(0.0–100.0)
State born (F)	Percent of Ca deaths	58	68	(0.0–100.0)
Current smoke (M)	Percent of adults	28.4	27.9	(20.6–37.0)
Current smoke (F)	Percent of adults	23.3	22.8	(15.0–34.4)

SMR: standardized mortality ratio, HS: high school, HHInc: household income, M: male, F: female, Ca: cancer; two outlier counties (Deer Lodge, MT; Storey, NV) were excluded.

**Table 2:** Stratified analysis of male and female lung cancer mortality by median groundwater arsenic level (*µ*g/L).

As Median	County Wt Males	Counties Males	Cases Males	SMR Males	95% CI Males	RR Males	County Wt Females	Counties Females	Cases Females	SMR Females	95% CI Females	RR Females
3.0	3.0	49	12,595	0.89	(0.84–0.94)	1.00	3.0	42	3,026	0.89	(0.81–0.96)	1.00
3.1–3.9	3.5	15	1,947	0.91	(0.82–0.99)	1.02	3.5	13	455	0.90	(0.77–1.03)	1.02
4.0–4.9	4.1	25	11,199	0.88	(0.82–0.94)	0.97	4.1	21	2,907	0.89	(0.81–0.96)	0.99
5.0–5.9	5.0	18	1,251	0.92	(0.81–1.04)	1.05	5.0	11	235	0.87	(0.73–1.02)	0.98
6.0–6.9	6.1	16	1,317	0.91	(0.79–1.02)	0.98	6.1	11	251	0.93	(0.82–1.05)	1.07
7.0–7.9	7.1	11	369	0.86	(0.70–1.01)	0.94	7.2	6	61	1.05	(0.80–1.30)	1.13
8.0–9.9	8.3	15	1,413	0.94	(0.85–1.03)	1.10	8.4	10	223	0.75	(0.58–0.92)	0.71
10.0–14.9	11.2	12	1,952	0.84	(0.73–0.94)	0.89	11.3	10	385	0.87	(0.72–1.02)	1.16
15.0–24.9	20.1	7	868	0.95	(0.78–1.12)	1.13	20.4	5	170	0.84	(0.57–1.10)	0.97
25.0–59.9	39.7	3	123	0.83	(0.53–1.13)	0.88	41.2	3	31	0.82	(0.67–0.97)	0.98

3.0	3.0	49	12,595	0.89	(0.84–0.94)	1.00	3.0	49	3,026	0.877	(0.81–0.96)	1.00
3.1–9.9	5.4	100	17,496	0.90	(0.82–0.99)	1.01	5.3	72	4,132	0.890	(0.84–0.94)	1.01
10.0–59.9	17.9	22	2,943	0.87	(0.82–0.94)	0.98	18.8	18	586	0.853	(0.76–0.95)	0.97

County Wt: county weighted, SMR: standardized mortality ratio, CI: confidence interval, RR: relative risk.

## References

[1] National Research Council (NRC) (1999). *Arsenic in Drinking Water*.

[2] Ferreccio C., González C., Milosavjlevic V., Marshall G., Sancha A. M., Smith A. H. (2000). Lung cancer and arsenic concentrations in drinking water in Chile. *Epidemiology*.

[3] Wu M.-M., Kuo T.-L., Hwang Y.-H., Chen C.-J. (1989). Dose-response relation between arsenic concentration in well water and mortality from cancers and vascular diseases. *American Journal of Epidemiology*.

[4] Cantor K. P. (2001). Invited commentary: arsenic and cancer of the urinary tract. *American Journal of Epidemiology*.

[5] United States Environmental Protection Agency (US EPA) (February 2010). Toxicological review of inorganic arsenic in support of summary information on the Integrated Risk Information System (IRIS). *Final Draft. Federal Register*.

[6] Lamm S. H., Ferdosi H., Dissen E. K., Li J., Ahn J. (2015). A systematic review and meta-regression analysis of lung cancer risk and inorganic arsenic in drinking water. *International Journal of Environmental Research and Public Health*.

[7] Buchet J. P., Lison D. (1998). Mortality by cancer in groups of the Belgian population with a moderately increased intake of arsenic. *International Archives of Occupational and Environmental Health*.

[8] Chen C.-L., Chiou H.-Y., Hsu L.-I., Hsueh Y.-M., Wu M.-M., Chen C.-J. (2010). Ingested arsenic, characteristics of well water consumption and risk of different histological types of lung cancer in northeastern Taiwan. *Environmental Research*.

[9] Dauphiné D. C., Smith A. H., Yuan Y., Balmes J. R., Bates M. N., Steinmaus C. (2013). Case-control study of arsenic in drinking water and lung cancer in California and Nevada. *International Journal of Environmental Research and Public Health*.

[10] Ferreccio C., Yuan Y., Calle J. (2013). Arsenic, tobacco smoke, and occupation: association of multiple agents with lung and bladder cancer. *Epidemiology*.

[11] Steinmaus C., Ferreccio C., Yuan Y. (2014). Elevated lung cancer in younger adults and low concentrations of arsenic in water. *American Journal of Epidemiology*.

[12] Han Y.-Y., Weissfeld J. L., Davis D. L., Talbott E. O. (2009). Arsenic levels in ground water and cancer incidence in Idaho: an ecologic study. *International Archives of Occupational and Environmental Health*.

[13] D’Ippoliti D., Santelli E., De Sario M. (2015). Arsenic in drinking water and mortality for cancer and chronic diseases in central Italy, 1990–2010. *PLOS ONE*.

[14] Heck J. E., Andrew A. S., Onega T. (2009). Lung cancer in a U.S. population with low to moderate arsenic exposure. *Environmental Health Perspectives*.

[15] García-Esquinas E., Pollán M., Umans J. G. (2013). Arsenic exposure and cancer mortality in a US-based prospective cohort: the strong heart study. *Cancer Epidemiology Biomarkers & Prevention*.

[16] Lamm S. H., Engel A., Kruse M. B. (2004). Arsenic in drinking water and bladder cancer mortality in the United States: an analysis based on 133 U.S. Counties and 30 years of observation. *Journal of Occupational and Environmental Medicine*.

[17] National Research Council (NRC) (2001). *Arsenic in Drinking Water, 2001 Update*.

[18] United States Environmental Protection Agency (US EPA) (2001). National Primary Drinking Water Regulations: arsenic and clarification to compliance and new source contaminants monitoring. *Final Rule. Federal Register*.

[19] Morales K. H., Ryan L., Kuo T.-L., Wu M.-M., Chen C.-J. (2000). Risk of internal cancers from arsenic in drinking water. *Environmental Health Perspectives*.

[20] Focazio M. J., Welch A. H., Watkins S. A., Helsel D. R., Horn M. A. (1999). A retrospective analysis on the occurrence of arsenic in ground-water resources of the united states and limitations in drinking-water-supply characterizations. *Water-Resources Investigations Report*.

[21] National Cancer Institute/US Environmental Protection Agency (NCI/EPA) (1983). US cancer mortality rates and trends 1950–1979.

[22] United States Census Bureau (2014). *Median Household Income by County: 1969, 1979, 1989. 1970, 1980, and 1990 Censuses of Population and Housing*.

[23] Minnesota Population Center (2011). *National Historical Geographic Information System (NHGIS): Version 2.0*.

[24] National Center for Health Statistics (U.S.) (1979). *Public Use Data Tape Documentation*.

[25] United States Geological Survey http://mrdata.usgs.gov/geochem/doc/averages/countydata.htm.

[26] Centers for Disease Control and Prevention (CDC) (2014). *Behavioral Risk Factor Surveillance System Survey Data (BRFSS)*.

[27] Dwyer-Lindgren L., Mokdad A. H., Srebotnjak T., Flaxman A. D., Hansen G. M., Murray C. J. L. (2014). Cigarette smoking prevalence in US counties: 1996–2012. *Population Health Metrics*.

[28] StataCorp (2009). *Stata Statistical Software: Release 11*.

[29] Microsoft (2010). *Microsoft Excel. Redmond*.

[30] SEER (2014). *Surveillance, Epidemiology, and End Results (SEER) Program Research Data (1973–2011)*.

[31] Welch K., Higgins I., Oh M., Burchfiel C. (1982). Arsenic exposure, smoking, and respiratory cancer in copper smelter workers. *Archives of Environmental Health*.

[32] Lamm S. H., Lederer W. H., Whipple C., Covello V. T. (1983). Inorganic arsenic: importance of accurate exposure characterization for risk assessment. *Risk Analysis in the Private Sector*.

[33] Peters S., Reid A., Fritschi L., Bill Musk A. W., de Klerk N. (2013). Cancer incidence and mortality among underground and surface goldminers in Western Australia. *British Journal of Cancer*.

[34] Lamm S. H., Robbins S., Chen R., Lu J., Goodrich B., Feinleib M. (2014). Discontinuity in the cancer slope factor as it passes from high to low exposure levels—arsenic in the BFD-endemic area. *Toxicology*.

[35] Beverage Marketing Corporation (BMC), http:earthpolicy.org/data center/C24 (2014). *Bottled Water Consumption in the United States, 1976–2007*.

[36] Pou S. A., Osella A. R., Diaz M. P. (2011). Bladder cancer mortality trends and patterns in Cordoba. Argentina (1986–2006). *Cancer Causes Control*.

[37] Ryker S. J. (2001). Mapping arsenic in ground water: a real need, but a hard problem. *Geotimes*.

[38] Xue J., Zartarian V., Wang S.-W., Liu S. V., Georgopoulos P. (2010). Probabilistic modeling of dietary arsenic exposure and dose and evaluation with 2003-2004 NHANES data. *Environmental Health Perspectives*.

[39] Kurzius-Spencer M., Burgess J. L., Harris R. B. (2014). Contribution of diet to aggregate arsenic exposures—an analysis across populations. *Journal of Exposure Science and Environmental Epidemiology*.

[40] Tao S. H., Bolger P. M. Dietary intakes of arsenic in the United States.

[41] National Research Council (NRC) (1999). *Arsenic in Drinking Water. National Academy of Sciences*.

[42] Smith D. B., Cannon W. F., Woodruff L. G., Solano F., Kilburn J. E., Fey D. L. http://pubs.usgs.gov/ds/801/.

[43] Putila J. J., Guo N. L. (2011). Association of arsenic exposure with lung cancer incidence rates in the United States. *PLoS ONE*.

[44] Agency for Toxic Substances and Disease Registry (ATSDR) (2007). *Toxicological Profile for Arsenic*.

[45] American Lung Association (ALA) http://www.lung.org/assets/documents/research/tobacco-trend-report.pdf.

[46] Gallup Tobacco and Smoking. http://www.gallup.com/poll/1717/tobacco-smoking.aspx.

[47] Chiou H. Y., Hsueh Y. M., Liaw K. F. (1995). Incidence of internal cancers and ingested inorganic arsenic: a seven-year follow-up study in Taiwan. *Cancer Research*.

[48] Tsuda T., Babazono A., Yamamoto E. (1995). Ingested arsenic and internal cancer: a historical cohort study followed for 33 years. *American Journal of Epidemiology*.

[49] Gebel T. W., Suchenwirth R. H. R., Bolten C., Dunkelberg H. H. (1998). Human biomonitoring of arsenic and antimony in case of an elevated geogenic exposure. *Environmental Health Perspectives*.

[50] Demir N., Enon S., Turksoy V. A. (2014). Association of cadmium but not arsenic levels in lung cancer tumor tissue with smoking, histopathological type and stage. *Asian Pacific Journal of Cancer Prevention*.

[51] Cohen S. M., Arnold L. L., Beck B. D., Lewis A. S., Eldan M. (2013). Evaluation of the carcinogenicity of inorganic arsenic. *Critical Reviews in Toxicology*.

[52] Snow E. T., Sykora P., Durham T. R., Klein C. B. (2005). Arsenic, mode of action at biologically plausible low doses: what are the implications for low dose cancer risk?. *Toxicology and Applied Pharmacology*.

[53] Lamm S. H., Engel A., Penn C. A., Chen R., Feinleib M. (2006). Arsenic cancer risk confounder in Southwest Taiwan data set. *Environmental Health Perspectives*.

[54] Lamm S. H., Robbins S. A., Zhou C., Lu J., Chen R., Feinleib M. (2013). Bladder/lung cancer mortality in blackfoot-disease (BFD)-endemic area villages with low (<150 *μ*g/L) well water arsenic levels—an exploration of the dose-response poisson analysis. *Regulatory Toxicology and Pharmacology*.

[55] Niedzwiecki M. M., Hall M. N., Liu X. (2013). A dose-response study of arsenic exposure and global methylation of peripheral blood mononuclear cell DNA in Bangladeshi adults. *Environmental Health Perspectives*.

[56] Karakulak U. N., Yilmaz O. H., Tutkun E. (2016). Evaluation of aortic elasticity parameters in arsenic exposed workers. *Journal of Human Hypertension*.

[57] World Health Organization (WHO) Safety evaluation of certain contaminants in food: safety evaluation of certain contaminants in food. http://apps.who.int/iris/bitstream/10665/44520/1/9789241660631_eng.pdf.

